# Joining Strategies for Double-Sided Self-Pierce Riveting

**DOI:** 10.3390/ma16031191

**Published:** 2023-01-30

**Authors:** Rafael M. Afonso, Luís M. Alves

**Affiliations:** IDMEC, Instituto Superior Técnico, University of Lisbon, Av. Rovisco Pais, 1049-001 Lisbon, Portugal

**Keywords:** joining technology, mechanical joining, lap joints, sheets, self-pierce riveting

## Abstract

Double-sided self-pierce riveting (DSSPR) has been presenting itself as a proper alternative to self-pierce riveting (SPR) with many advantages for joining geometries of different thicknesses and cross-sections. To ensure its successful future industrial application, this paper presents a detailed comparison between different strategies to produce mechanical joints by means of the DSSPR process and discusses its performance and feasibility. Results show that the use of flat-bottom holes in both sheets provide interesting results, since they allow for a precise positioning of the tubular rivet in specific pre-defined locations, thus avoiding an incorrect joining procedure. This strategy tightens the tolerances of the process, while keeping a suitable level of destructive performance as demonstrated by the lap shear tests. Pre-riveting of the sheet has also been shown to produce suitable results in combination with or without a flat-bottom hole in the opposite sheet. This strategy comes at a cost of a slightly lower performance than that obtained with flat-bottom holes in both sheets, although the requirements of force and energy to complete the joining process are smaller. The conclusions of this research work are essential for selecting the joining strategy with DSSPR according to the requirements of the intended application.

## 1. Introduction

**Figure 1:**
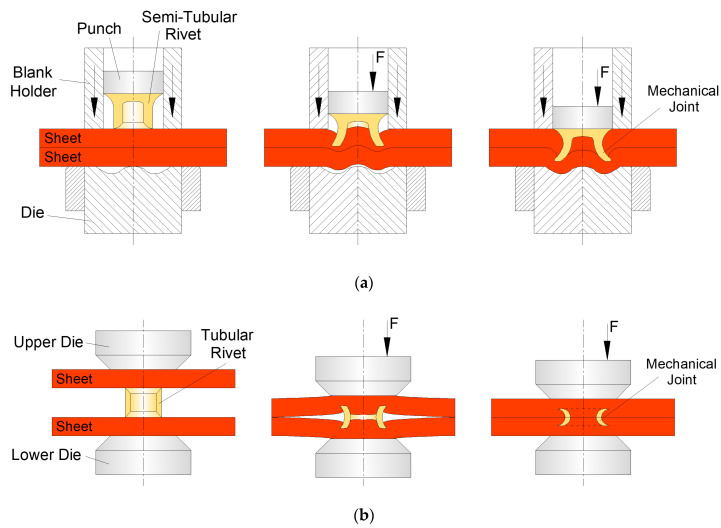
Schematic representation of the process sequence for joining sheets by means of (**a**) self-pierce riveting and (**b**) double-sided self-pierce riveting.

For the production of mechanical joints by means of the technology of joining by forming with auxiliary joining elements [1], self-pierce riveting (SPR) has been the preferred mechanical joining technology over the years to produce mechanical interlockings between two or more geometries to be joined [2]. Form-fit joints are therefore created by employing those semi-tubular rivets as unremovable mechanical fasteners (Figure 1a).

Lightweight construction demands for multi-material design, which in turn demand the utilization of versatile joining processes that circumvent the metallurgical incompatibilities, provide a low heat input and can provide an adequate level of flexibility [3]. At the same time, a versatile process chain required for product manufacturing with this kind of joining by forming processes [4] encompasses the material combination (joining suitability), the design and layout of the joints (joining safety), and the adaptability and predictability of the joining process (joining possibility). Thus, it becomes necessary to reduce the amount of joining parameters and configurations, as well as the tool variants, that depend on each material combination, and present themselves as the limitations of conventional self-pierce riveting [5].

The application of SPR to aluminium sheets of different alloys have been extensively investigated. From those studies, the effect of rivet and die shapes on the joint properties have been studied [6,7,8]. The analysis of the influence of the sheet thickness on the fatigue performance of aluminium SPR joints [9] concluded that the fatigue life of the SPR joints was extended with larger sheet thicknesses, where failure tends to occur at the bottom sheet along the joint button. In addition, the joints made from thick aluminium sheets have good mechanical stability, whereas the joints made from thin aluminium sheets have poor durability in corrosive environments [10]. For the same aluminium alloy utilized in this research, the influence of the surface conditions on the SPR joint strength was assessed [11], from which it was concluded that the increase of the surface roughness allows us to increase the shear strength of the SPR lap joints. Different strategies have been developed to accommodate the joining of multi-material structures and advanced materials [12]. The corresponding joint failure mechanisms under different mechanical loading conditions [13,14] and the joint corrosion issues [14] have been carefully analysed, from which it can be concluded that there is still room for improvement.

An innovative self-pierce riveting process consists of double-sided self-pierce riveting (DSSPR) which makes use of a tubular rivet with chamfered ends that are capable of producing hidden joints between sheets placed over each other (Figure 1b). The tubular rivets are placed in-between the sheets to be joined and forced through them, while their ends are flared to create a mechanical interlocking [15,16]. In comparison with SPR, some important advantages are presented by DSSPR such as: the ability to join different thicknesses without limitations for larger thicknesses since is not necessary to tear up the pierced sheet as it happens for SPR; the reduced to non-existent material protrusions in the sheet surfaces; smaller levels of controlled deformation and reduced damage and stress–strain levels in the materials; the joint not being exposed to galvanic corrosion or other elements; the simpler geometry of the tubular rivet and process parameters; dedicated tools no longer being needed other than flat compression plates.

The validation of the DSSPR joining technology has been performed with tubular rivets of stainless steel AISI 304 and aluminium AA5754 sheets, to investigate the working principle and the geometric scalability of the tubular rivets. Recently, the chamfered ends of the rivets have been optimized to improve the rivet penetration and final morphology, and therefore increase the overall joint strength of the mechanical connection [17].

Since there is no upper thickness limit in DSSPR contrary to what it is observed for SPR, sheets with a thickness of 5 mm were originally tested [16], and more recently, sheets with a thickness of 1.5 mm were investigated [18]. From this last study, it was concluded that the external diameter of the tubular rivet can remain constant, although its height and thickness need to be modified in order to match the thickness of the sheet. This means that the initial rivet thickness needs to be lower than the smaller sheet thickness and the initial rivet height needs to be at least equal to double the smaller sheet thickness, in order to produce a proper mechanical interlocking.

From an industrial point of view or when demanded from the material combination, some strategies may need to be developed. For instance, the authors have shown that while keeping all the other parameters constant, the chamfered angle of the tubular rivet can be modified at each end of the rivet, to account for the different resistances to penetration of the materials from the two sheets [19]. However, when those differences are too high (for example, the combination of a PVC sheet with an Aluminium sheet), an asymmetric and reduced mechanical interlocking is formed at the harder sheet material. For those situations, another strategy is then presented in that same work [19] to produce a symmetrical joint with a good mechanical interlocking in both sheets: the tubular rivet is pre-riveted in the harder sheet by a dedicated compression tool and then subsequently pressed against the softer sheet to produce the joint between the two sheets. This solution allows us to keep the same chamfered angle of the rivet in both sides, thus avoiding possible errors during the positioning of the rivet, and also allowing us to join sheets with very different mechanical resistances, which is a step forward towards an adequate industrial implementation.

Another possible solution is the introduction of flat bottom holes in the strongest sheet by means of machining or forming, in order to correctly position the tubular rivets before piercing. This promotes the penetration in harder materials and their subsequent assembly to softer materials by means of the opposite end of the tubular rivet [20]. At the same time, the excess volume of sheet material during the rivet penetration will flow through the empty spaces of the flat-bottom hole, thus eliminating any protrusion above the sheet surfaces. In terms of industrial implementation, this solution allows us to easily replicate and inspect the joining process, since the position of the rivet is well-defined and no additional joining stages or changes to the process parameters are needed. This way, it is possible to join different materials in one single joining operation while the rivet is secured in the correct position, which is essential when the sheets to be joined are not horizontal.

Therefore, the objective of this work is to combine the different previous strategies and compare their performance when subjected to static loads. To support the investigation, numerical predictions are employed to analyse the mechanics of the deformation and the stress–strain levels for the different strategies and modifications introduced. Along with the numerical analysis, different specimens are produced and subjected to lap shear strength tests to determine the performance produced by each modification. This will allow us to define the strategy to be followed in accordance with the load and energy requirements for both the joining process and the intended application, towards a closer industrial implementation of DSSPR. Different compromises are made with each joining strategy, although the introduction of flat-bottom holes in both sheets is able to provide a similar performance to conventional DSSPR joints without holes, that offers the best performance among all strategies. The utilization of pre-riveting follows the introduction of flat-bottom holes in terms of performance and its applicability regards the utilization of sheets of very different strengths or thicknesses.

## 2. Materials and Methods

### 2.1. Material Flow Curves

**Figure 2:**
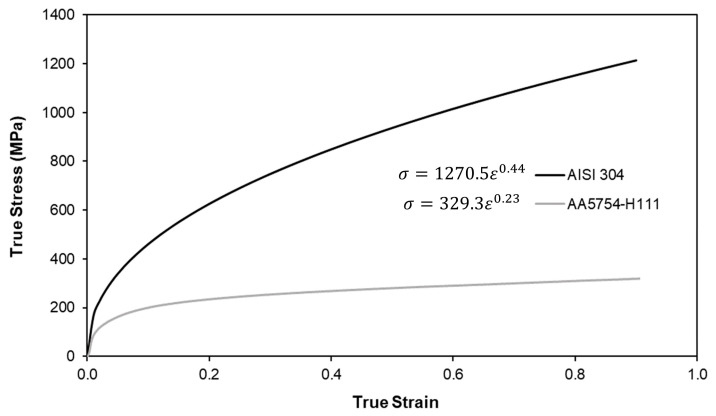
Flow curves of the AISI 304 stainless steel rivets and of the AA5754-H111 aluminium sheets. Each respective Ludwik–Holomon equation is also presented.

The materials chosen and their respective flow curves were retrieved from a previous work of the authors on conventional DSSPR [16] to which a comparison is to be made when discussing the performance of the joining process. Effectively, commercial AA5754-H111 aluminum sheets with 5 mm thickness and AISI 304 stainless steel tubes with an outer diameter of 10 mm and 1.5 mm wall thickness were utilized. These materials will be applied to the different strategies that were until now only tested with dissimilar material combinations. The flow curves involved tensile and stack compression tests in a hydraulic testing machine (Instron SATEC 1200 kN, Norwood, MA, USA) with a speed of 5 mm/min, whereas the flow curve of the AISI 304 tubes was determined by means of tensile tests that were carried out in the same experimental testing conditions. The respective flow curves were approximated by means of a power-law hardening model (Ludwik-Holomon) and are presented in Figure 2.

### 2.2. Work Development

**Figure 3:**
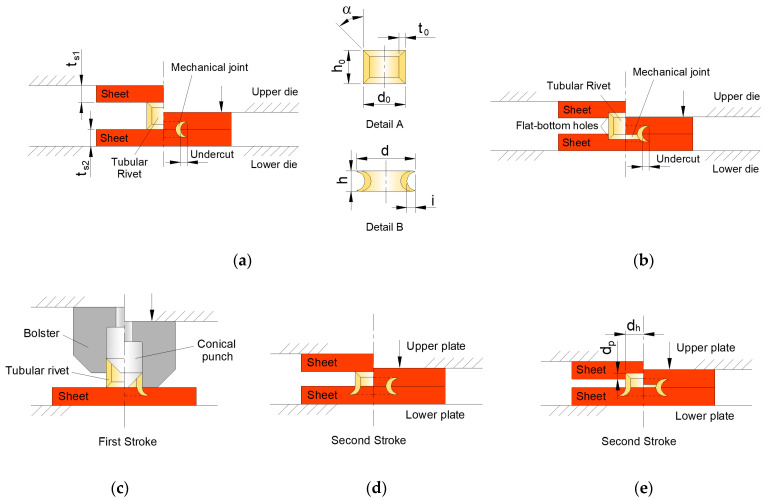
(**a**) Conventional DSSPR with notation of the process parameters and details of the tubular rivet in each stage, (**b**) DSSPR with a flat-bottom hole in each sheet, and (**c**) pre-riveting of one sheet and subsequent assembly to a (**d**) plain sheet or (**e**) a sheet with a flat-bottom hole.

The main process parameters of the tubular rivet were retrieved from the original work on conventional DSSPR [16] and resume itself to those parameters highlighted in Figure 3 that were kept constant along the work: (i) the outer diameter $d_{0}$, (ii) the height $h_{0}$, (iii) the wall thickness $t_{0}$, and (iv) the chamfered angle α of the rivet ends (refer to the details in Figure 3a).

Regarding the sheets, the upper and lower sheet thicknesses $t_{s}$ remained constant, while for sheets with a flat-bottom hole, the diameter of the hole $d_{h}\;$was kept constant and the influence of the depth $d_{p}$ of the flat-bottom hole (Figure 3b) was evaluated. The dedicated compression tool consisting of a bolster and a conical punch to force the tubular rivet through one of the sheets (Figure 3c), that will then be pressed through the opposite sheet without (Figure 3d) or with a flat-bottom hole (Figure 3e), was taken from a previous work by the authors [19].

The different strategies consist of:

1. Conventional DSSPR between two plain sheets;
2. DSSPR between sheets with each one having a flat-bottom hole with a given depth $d_{p}$;
3. DSSPR with pre-riveting of one of the sheets in combination with a plain sheet;
4. DSSPR with pre-riveting of one of the sheets in combination with a sheet having a flat-bottom hole with a given depth $d_{p}$;

The experimental work was carried out at ambient temperature in the same hydraulic testing machine utilized to previously obtain the material flow curves. The range of values for each parameter is presented on Table 1. 

A minimum of five specimens were produced for each strategy with some being halved lengthwise to observe and compare the mechanical interlocking i obtained in each mechanical joint, while the remaining specimens were subjected to destructive lap shear tests to evaluate and compare the performance of the joints obtained by the different strategies.

### 2.3. Numerical Simulations

The numerical simulations of DSSPR were performed with the finite element computer program i-form [21] where the previous works on DSSPR were already validated. This program is based upon the flow formulation, which is built in accordance with the weak form of quasi-static force equilibrium modified to include the contact and sliding with friction between deformable objects, according to the equation:

(1) $$\int_{V}\sigma_{ij}^{\prime }\delta D_{ij}dV+\int_{V}\sigma_{m}\delta D_{v}dV-\int_{S_{t}}t_{i}\delta u_{i}dS+\int_{S_{f}}\left(\int_{0}^{\left|u_{r}\right|}\tau_{f}\delta u_{r}\right)dS+K_{1}\overset{N_{c}}{\underset{c=1}{\sum }}g_{n}^{c}\delta g_{n}^{c}=0$$

The finite element equations resulting from (1) use a control volume with velocities $u_{i}$ as the primary unknowns and are written in the current (deformed) configuration, following a modified ‘updated Eulerian’ approach [22]. The symbols included in (1) are related to the deviatoric Cauchy stress $\sigma_{ij}^{\prime }$, the hydrostatic stress $\sigma_{m}$, the rate of deformation $D_{ij}$, and the volumetric rate of deformation $D_{v}$, which make the overall computer implementation similar to that of a viscous fluid subjected to relaxation of the incompressibility condition of the velocity field $D_{v}=0$ by means of a penalty function K, with $\sigma_{m}=\left(K/2\right)D_{v}$. Other symbols in (1) denote the tractions $t_{i}$ applied on the boundary $S_{t}$ of the control volume and the friction shear stress $\tau_{f}$. The relative sliding velocity $u_{r}$ acting on the contact interfaces $S_{f}$ between the deformable and rigid objects is also denoted. The contact between deformable objects by means of a two-pass contact search algorithm is accounted in the last term of Equation (1). The symbols $N_{c}$ and $g_{n}^{c}$ denote the contact pairs and the corresponding normal gap velocities, which are penalized by a large number $K_{1}$ to avoid penetration, as it is comprehensively explained in [21].

**Figure 4:**
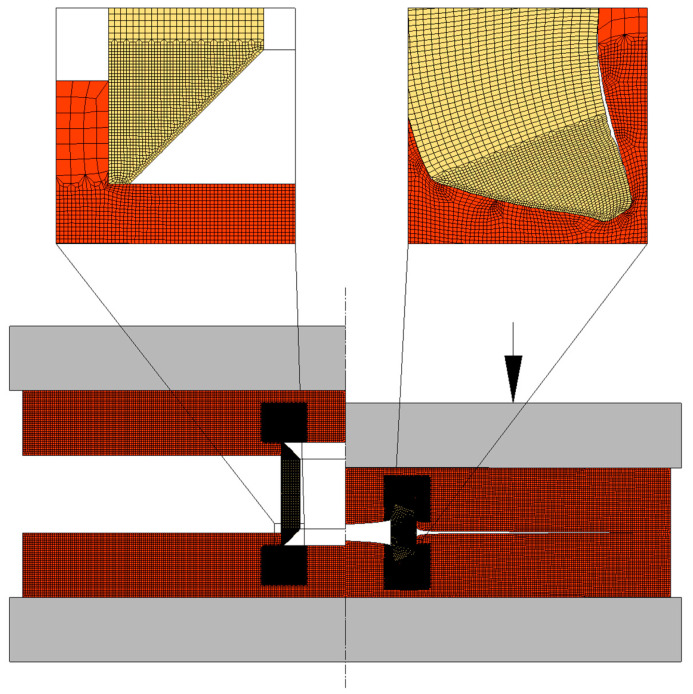
Finite element model at the start and end of the DSSPR joining process of two sheets having flat-bottom holes with a depth $d_{p}$ of 1 mm.

A rotational symmetry was considered for modelling the plastic deformation, with the sheets and tubular rivets being modelled as deformable isotropic objects subjected to axisymmetric loading. Their cross-sections were discretized by means of quadrilateral elements with a larger number of elements at the locations where the riveting process takes place and larger deformations are generated (refer to Figure 4). The tools were modelled as rigid objects and their contours were discretized by means of linear friction elements.

Regarding the friction conditions, a friction factor of m equal to 0.1 was selected for the contact interfaces between the deformable and rigid objects, whereas for the contact between deformable objects, a friction factor of m equal to 0.3 was utilized in accordance with the material combination of stainless steel and aluminium. Under these friction conditions, a strong correlation between the predicted numerical and experimental forces was observed.

Along the simulations, the finite element flow formulation equilibrium is checked by means of an iterative procedure meant to minimise the residual of (1) to within a specified tolerance. A convergence criterion of the residual equal to 10^−3^ was employed after which the geometry was updated based on the calculated velocities. Whenever large local deformations were generated from the rivet being pushed through the sheets which distorted some mesh elements, local repairment of the finite element model was carried out several times by semi-automatic repositioning of nodal points with appropriate transfer of field variables from previous to newer locations. That procedure was in some cases complemented by intermediate global remeshings of the entire deformed objects. 

## 3. Results

Comparisons between the Different Strategies

**Figure 5:**
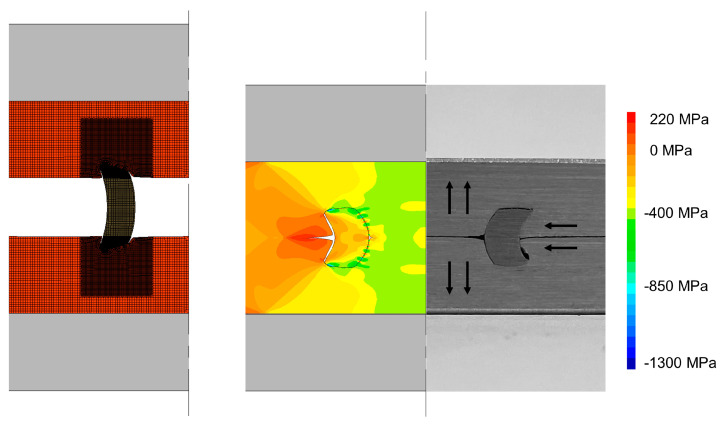
Evolution of conventional DSSPR with notation of the direction of material flow after elastic recovery of the materials. The distribution of radial stress $\sigma_{r}$ is presented.

Through the combination of finite element modelling and experimentation, it was possible to identify the mechanics of deformation for the different strategies analysed. Generally, the thickness of the tubular rivet increases along the deformation due to compression and strain hardening of the sheet and rivet, the latter being responsible for promoting a combined piercing and flaring of the tubular rivet, which results in the formation of a mechanical interlocking [16]. As the rivet penetrates through the sheets, the sheet material flows over the rivet to accommodate the volume of the rivet being pushed into the sheets. For plain sheets (Figure 5), the material flow is very constrained and as a result the stress levels increase as well as the joining force.

After the tools were removed, a very small protrusion is visible in the top of the sheet surfaces which results from the elastic recovery of the materials from those sheets (refer to the arrows in the photograph in Figure 5) in the opposite direction through which they were forced while the rivet was pushed through them. This causes the rivet ends to curl for compensating the constraint caused by the strong contact between the two overlapped sheets at the centre of the joining region.

**Figure 6:**
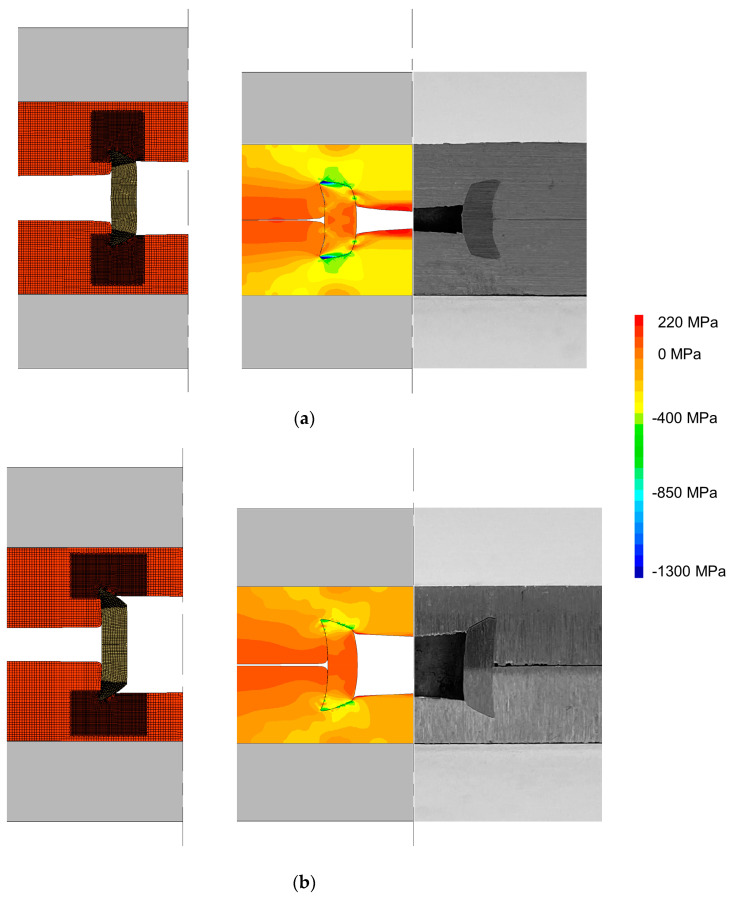
Evolution of DSSPR for sheets with flat-bottom holes with depths $d_{p}$ of (**a**) 1 mm and (**b**) 2 mm. The respective radial stress distribution is presented.

To overcome the constraints in material flow, flat-bottom holes were introduced in the sheets to allow for positioning and aligning both the rivet and the two sheets to be joined. As seen in Figure 6, which discloses the experimental and finite element predicted cross-sections of two joints with flat-bottom holes of different depths $d_{p}$, the amount of unfilled volume between the outer rivet wall and adjacent sheet material (that is normally observed for conventional DSSPR) is significantly reduced.

The gap created by the flat-bottom holes allows us to reduce the upward elastic recovery movement, which in turn results in even smaller protrusions than those created by conventional DSSPR. This happens because the pressures at the sheet surfaces are highly reduced with the introduction of the holes and the sheet material can flow towards the inside of the rivet diameter without making contact with the opposite sheet. The resulting form-closed joining mechanism is now responsible for a smaller mechanical interlocking than in conventional DSSPR, although a stronger force-closed mechanism develops at the contact interface between the rivet and the flat-bottom hole due to the residual normal pressures after the unloading of the tools. Depending on the depth $d_{p}$ of the flat-bottom hole, the radial stresses developed will prevent tangential movement due to friction and may produce higher mechanical performances than other strategies, as it will be seen in Section 4.

The finite-element-predicted distributions and experimental results in Figure 6 allow us to discuss the plastic deformation for different variations of the depth $d_{p}$. For a depth $d_{p}\;$= 1 mm (Figure 6a), the rivet is more restrained by the material from the sheet which will provide a sounder joint that is able to support larger tangential movements due to the development of larger levels of radial stress. Nevertheless, it was previously seen by the authors [20] that, for sheets made from materials of very different mechanical strengths, these smaller depths may result in the rivet being mainly pierced through the softer sheet without producing a proper mechanical interlocking if the chamfered angle of the rivet is not properly controlled. A larger depth $d_{p}$ of 2 mm (Figure 6b) is not able to offer enough constriction between the rivet and the sheets, which will result in the rivet detaching more easily from the sheets as it is forced to unbend during its lap shear destruction. The radial pressures are very low in comparison with a depth $d_{p}$ of 1 mm which demonstrate the lack of rivet penetration into the sheets. Therefore, a larger increase of the depth $d_{p}$ will not bring any advantages and may compromise the integrity of the sheet while limiting the industrial application of this strategy for smaller thicknesses.

**Figure 7:**
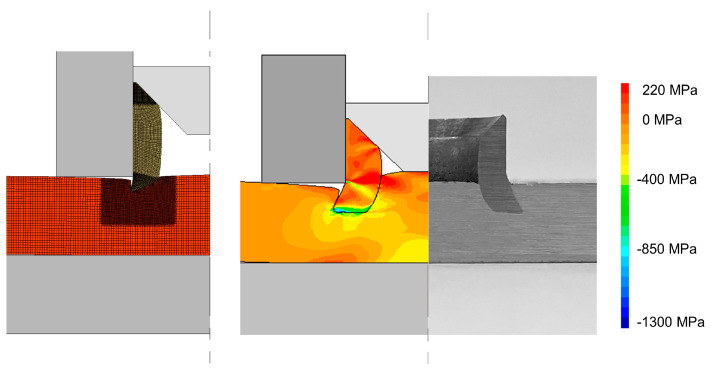
Pre-riveting of a plain sheet at intermediate and final stages with the correspondent photograph and distribution of radial stress.

Regarding the strategy of pre-riveting the rivet in one of the sheets, a dedicated compression tool forces the tubular rivet into the sheet and creates a mechanical interlocking between those two elements, as seen in Figure 7.

Then, the other sheet is placed over the opposite free end of the tubular rivet and compressed until a point where the sheets contact with each other, and the joint is produced. Generally, the pre-riveting should be applied to the harder sheet and/or thicker sheet in order to guarantee an adequate penetration of that sheet, since after the pre-riveting operation, the already joined and strain-hardened region will provide a better resistance to deformation and force it to occur mostly at the free region of the opposite rivet end.

**Figure 8:**
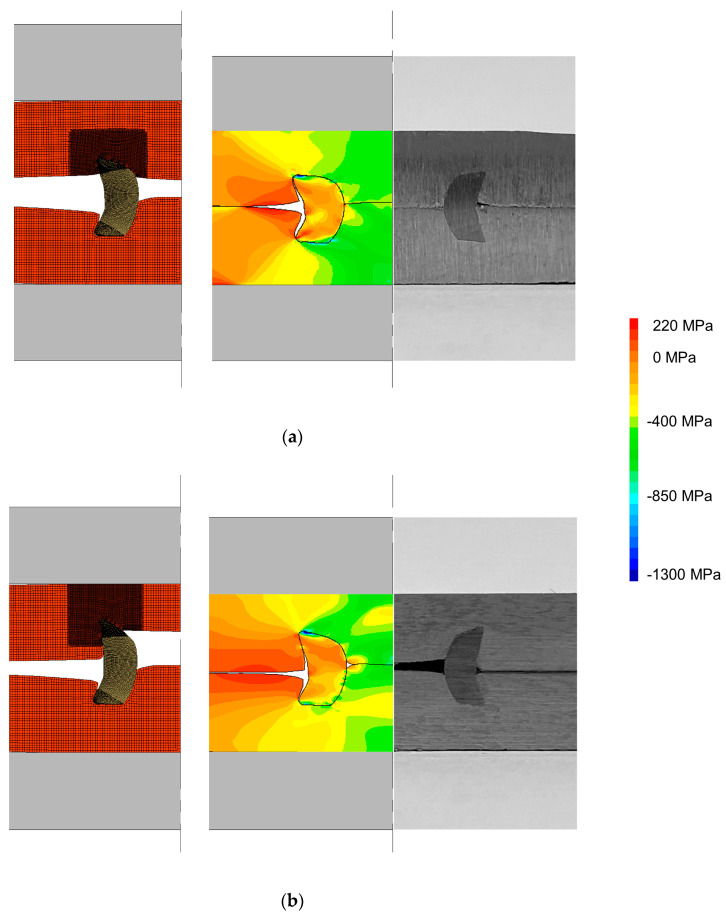
Evolution of DSSPR when joining a pre-riveted lower sheet to an upper sheet with (**a**) and (**b**) without a flat-bottom hole.

Two modifications were analysed for the pre-riveting strategy: one of them consists of joining the pre-riveted sheet with a plain sheet (Figure 8a), while the other consists of joining the pre-riveted combination with a sheet having a pre-drilled hole with a depth $d_{p}$ = 1 mm (Figure 8b), that, as seen before, is able to improve the sheet material constraint around the tubular rivet. In both cases, the radial pressures to which the pre-riveted sheet was subjected increased mainly at the inner rivet diameter, where the sheets were compressed against each other (refer to the comparison between Figure 7 and Figure 8).

In comparison with the results previously obtained without the pre-riveting stage, the mechanical interlockings are now larger (0.699 mm for the case of the plain sheet and 0.521 mm for the sheet with a depth $d_{p}$ of 1 mm), mainly due to the fact that the other two elements (rivet and pre-riveted sheet) will act as a single element and their strain-hardened material regions will concentrate the deformation in the opposite ends of the rivet where the joining with the sheet occurs (refer to the upper sheet in Figure 8a,b). Despite the differences in the values of mechanical interlocking and in the joint morphology of these two variations with a pre-riveting stage, the distribution of radial stress is similar, which may justify the similarity of their destructive performances, as it will be later seen.

Overall, the agreement between the finite element predictions and their respective experimental specimens is suitable for all the different strategies. Minor variations are attributed to small differences in dimensions resulting from the manufacturing process of the different geometries and/or to the elastic recovery of the materials after being cut lengthwise to reveal the cross-section of the joint, and allow a comparison between the numerical and experimental results.

## 4. Discussion

### 4.1. Joining Forces

**Figure 9:**
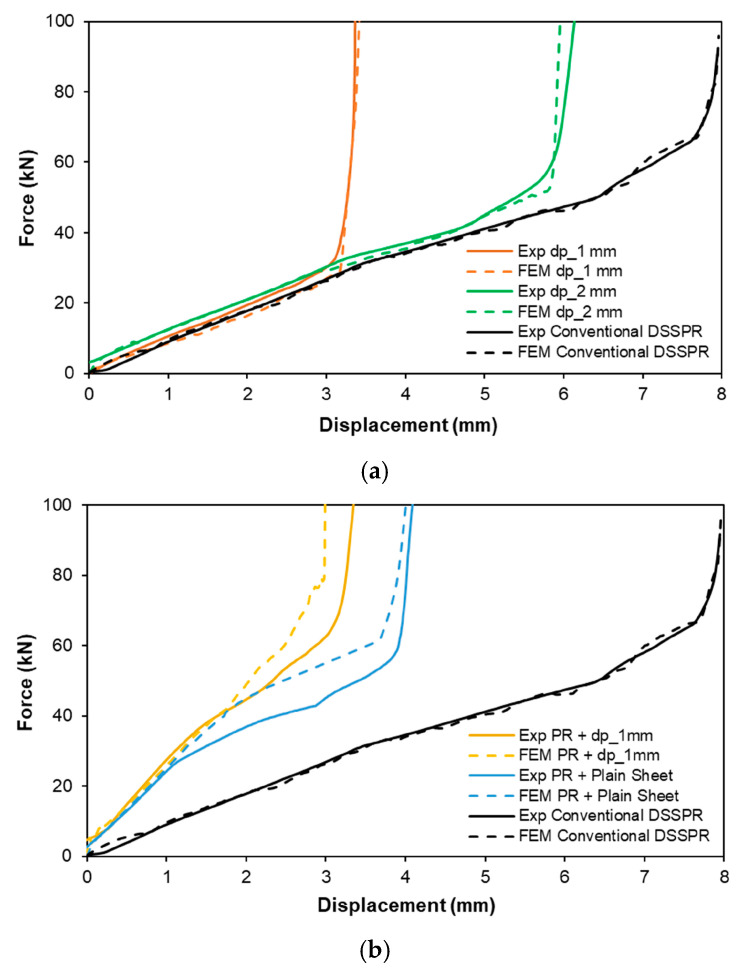
Force-displacement evolutions of the different cases obtained by means of numerical and experimental testing for (**a**) sheets with a flat-bottom hole in each sheet and (**b**) with an initial pre-riveting operation. The results from conventional DSSPR are included for reference.

The different strategies followed for the implementation of DSSPR gave rise to differences in the force-displacement evolutions shown in Figure 9, although all of them are characterized by an indentation stage, a combined piercing and flaring stage, a clamping stage, and a final overload stage, as verified for conventional DSSPR [16].

The forces to clamp the two sheets together are the maximum for the cases where plain sheets are employed (conventional DSSPR and pre-riveting on two plain sheets) to which it follows a steep increase of the overall force as the two overlapped sheets are being pressed against each other. The required maximum force to produce the mechanical joints is in the range of 100 kN for all the strategies analysed.

Conventional DSSPR demands higher levels of joining energy, as the total rivet height has to penetrate the two plain sheets. In contrast, smaller levels of joining energy are found to the other alternatives, in particular for the alternative of sheets with flat-bottom holes, since the joining energy for the pre-riveted joints is the sum of the energy in each one of the two stages.

The differences in displacement for the different joining strategies in comparison with conventional DSSPR are due to the smaller free height of the rivets that is now placed inside the flat-bottom holes, whereas for the case of the pre-riveting strategy, only half of the rivet height is compressed against the sheet in each stage.

### 4.2. Destructive Performance Tests

**Figure 10:**
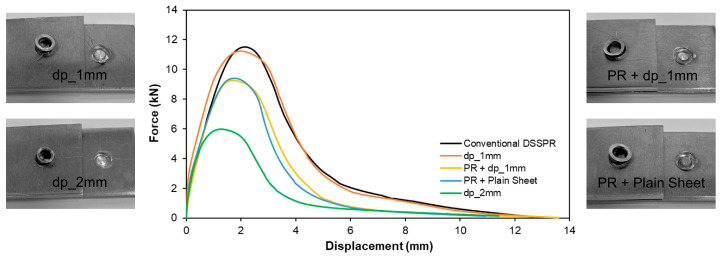
Evolution of force with displacement for the shear tests of the different modifications of DSSPR with the corresponding photographs of the specimens after detachment.

Regarding the destructive performance evaluation, the lap shear tests in Figure 10 allow us to conclude that the case with two sheets having flat-bottom holes with a depth $d_{p}$ of 1 mm provide the best compromise both in terms of joining force and energy (refer to Figure 9a), as well as in the force that the joint can withstand before failure occurs that is very similar to conventional DSSPR. It is worth mentioning that the free gap created by the introduction of the flat-bottom hole allows the sheet material to flow towards that region and around the rivet, without generating a small protrusion in the top of the sheet surfaces after elastic recovery of the materials involved in the joint, as observed for conventional DSSPR.

In contrast, the increase of the depth of the flat-bottom holes to 2 mm provided a lower performance, justified by the reduced penetration of the rivet that results from the utilization of a deeper flat-bottom hole. This facilitates the detachment of the rivet from the sheets, as seen by the photographs included in Figure 10, where in comparison with a penetration depth of 1 mm, the sheet at the hole region is barely deformed.

For the pre-riveted joints and despite the differences in the amount of mechanical interlocking, the performances are very similar whether a plain sheet or a sheet with a flat-bottom hole with a depth of 1 mm is employed, since the detachment is constrained by the larger strain hardening levels of the sheet material at the pre-riveted side. The photographs in Figure 10 that refer to these two pre-riveted cases also show similar deformation levels in the sheets at the previously joined region.

## 5. Conclusions

Different strategies were analysed to produce joints in overlapped sheets with DSSPR, making use of the strong advantages of this joining technology while ensuring the conditions for its industrial implementation in a wide range of scenarios.

The introduction of flat-bottom holes in the sheets ensures both positioning and alignment of the rivets, while eliminating any protrusions above the sheet surfaces. As a result of the gap created by the holes, the joining forces and energies are lower than for conventional DSSPR, while their performances are very similar. However, the depth of the flat-bottom holes cannot be so high because it can compromise the performance of the mechanical connection or may even not be feasible for thinner sheet thicknesses. In the latter case, pre-riveting of one of the sheets (generally the harder and/or thicker sheet) should be employed instead to ensure a proper riveted joint.

For the pre-riveting strategy, two iterations can be utilized without any relevant differences in terms of performance, other than the advantages that arise from the simplicity of placement of the opposite rivet end when a localized flat-bottom hole is already present in the opposite sheet. Nevertheless, if the opposite sheet material is much softer than the pre-riveted sheet material, a flat-bottom hole may create a weaker region in the softer sheet that can compromise the joining process. The selection of the pre-riveting strategy comes at a cost of a slightly reduced destructive performance and the need to have an additional stage other than the single stroke in which the other strategies are produced. Therefore, this strategy is more suited for different material and thickness combinations where a greater or lesser penetration of the rivets into the sheets may be desired.

In conclusion, the guidelines established during this work help to create the conditions for selecting a suitable joining strategy with DSSPR depending on the material and geometry specifications.

## Figures and Tables

**Table 1:** Operating process parameters utilized in the experimental and numerical evaluation of different possible strategies of DSSPR (refer to Figure 3).

Rivet				
Material	$d_{0}$ (mm)	$h_{0}$ (mm)	$t_{0}$ (mm)	α (º)
AISI 304	10	8	1.5	45
**Sheets**				
Material	$t_{s}$ (mm)	$d_{p}$ (mm)	$d_{h}$ (mm)	
AA5754-H111	5	1, 2	10	

## Data Availability

Not applicable.
